# Genome-Wide Analysis of the *TCP* Transcription Factor Genes in *Dendrobium catenatum* Lindl.

**DOI:** 10.3390/ijms221910269

**Published:** 2021-09-24

**Authors:** Li Zhang, Cheng Li, Danni Yang, Yuhua Wang, Yongping Yang, Xudong Sun

**Affiliations:** 1 The Germplasm Bank of Wild Species, Kunming Institute of Botany, Chinese Academy of Sciences, Kunming 650201, China; zhangli1@mail.kib.ac.cn (L.Z.); licheng@mail.kib.ac.cn (C.L.); yangdanni@mail.kib.ac.cn (D.Y.); wangyuhua@mail.kib.ac.cn (Y.W.); 2 University of Chinese Academy of Sciences, Beijing 100049, China

**Keywords:** *Dendrobium catenatum* Lindl., TCP transcription factor family, phylogenetic analysis, expression profiles, phytohormone response

## Abstract

Teosinte branched1/cycloidea/proliferating cell factor (*TCP*) gene family members are plant-specific transcription factors that regulate plant growth and development by controlling cell proliferation and differentiation. However, there are no reported studies on the *TCP* gene family in *Dendrobium catenatum* Lindl. Here, a genome-wide analysis of *TCP* genes was performed in *D. catenatum*, and 25 *TCP* genes were identified. A phylogenetic analysis classified the family into two clades: Class I and Class II. Genes in the same clade share similar conserved motifs. The GFP signals of the DcaTCP-GFPs were detected in the nuclei of tobacco leaf epidermal cells. The activity of *DcaTCP4*, which contains the miR319a-binding sequence, was reduced when combined with miR319a. A transient activity assay revealed antagonistic functions of Class I and Class II of the TCP proteins in controlling leaf development through the jasmonate-signaling pathway. After different phytohormone treatments, the *DcaTCP* genes showed varied expression patterns. In particular, *DcaTCP4* and *DcaTCP9* showed opposite trends after 3 h treatment with jasmonate. This comprehensive analysis provides a foundation for further studies on the roles of *TCP* genes in *D. catenatum*.

## 1. Introduction

The TCP transcription factor family was designated on the basis of its original identified members, *TEOSINTE BRANCHED1* (*TB1*) in maize (*Zea mays*) that functions to maintain apical dominance [1], *CYCLOIDEA* (*CYC*) in snapdragon (*Antirrhinum majus*) that controls floral symmetry [2], and *PROLIFERATING CELL FACTORS 1/2* in rice (*Oryza sativa*) that is associated with the cell cycle, regulates DNA replication and repair, maintains chromosomal morphology and structure, and controls chromosomal segregation [3,4]. The family members contain a highly conserved non-canonical basic helix–loop–helix motif that is involved in DNA-binding and protein–protein interactions. It occurs in 59 amino acids at the N-terminus within the designated TCP domain [5]. The TCP family members are classified into two subfamilies on the basis of their TCP domains, Class I (PCF or TCP-P class) and Class II (TCP-C class), and the members of class II are divided into the CIN and CYC/TB1 [5,6,7]. Compared with Class II, Class I members have 4-aa deletions in their TCP domains. In addition, some members of Class II have an arginine-enriched motif of 18 to 20 aa residues, referred to as an R domain, and an ECE motif, which is composed of a glutamic acid-cysteine-glutamic acid stretch.

The TCP transcription factor family has been identified in many plant species. For example, *Arabidopsis thaliana* has 24 *TCP* genes, *Oryza sativa* has 28 *TCP* genes, *Lycopersicon esculentum* has 30 *TCP* genes, *Populus euphratica* has 33 *TCP* genes, *Populus trichocarpa* has 36 *TCP* genes, *Citrullus lanatus* has 27 *TCP* genes, *Prunus mume* has 19 *TCP* genes, and *Petunia inflata* has 34 *TCP* genes [5,8,9,10,11,12]. The *TCP* genes have a variety of functions, participating in the regulation of numerous growth and developmental processes, such as the coordination of cell proliferation, inflorescence development, shoot branching, leaf development, phytohormone biosynthesis, and the circadian clock regulation [13,14,15,16,17,18,19,20,21,22,23,24].

*Dendrobium catenatum* Lindl. belongs to the Orchidaceae family, and it is a rare and precious Chinese herbal medicinal plant. It is mainly distributed in Southeast, South, and Southwest China, and it usually grows on cliffs to receive sufficient sunlight and adequate water in the wild [25,26]. Here, we identified 25 *TCP* genes in *D. catenatum* and analyzed their phylogenetic relationships, gene architectures, conserved domain profiles, and subcellular localizations. Furthermore, the expression levels of *DcaTCP* genes after different phytohormone treatment were examined.

## 2. Results

### 2.1. Identification of TCP Family Genes in D. catenatum

**Figure 1:**
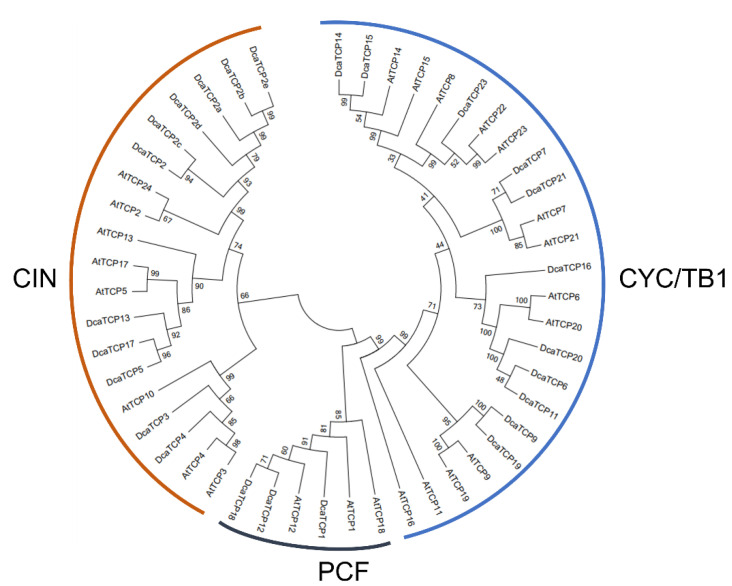
Phylogenetic tree of TCP proteins from *D. catenatum* and *Arabidopsis*. The phylogenetic tree was generated using the neighbor-joining (NJ) method implemented in the MEGA 7.0 software with JTT model and the pairwise gap deletion option. Bootstrap analysis was conducted with 1000 iterations.

The release of the complete *D. catenatum* genome allowed the genome-wide identification of genes. In the present study, BLAST searches were used to identify *DcaTCP*s in the *D. catenatum* genome. The obtained sequences were further verified using the HMM in SMART and Pfam. In total, 25 TCP-like sequences that possessed the conserved TCP motif were identified from the *D. catenatum* genome. The *DcaTCP* genes were annotated following the nomenclature of *A. thaliana* in accordance with protein sequence similarities (Figure 1).

The DcaTCP proteins were classified into two subclades (subclade PCF within Class I, and subclades CYC/TB1 and CIN within Class II). Among them, 11 and 14 DcaTCPs clustered into Class I (PCF) and Class II, respectively. A sequence analysis revealed that AtTCP8, -10, -22, and -24 had no orthologs in *D. catenatum*, whereas AtTCP2 had more than one ortholog in the genome.

The lengths of the DcaTCPs ranged from 178 (DcaTCP1) to 450 (DcaTCP2b) aa, the molecular weights ranged from 20.03 (DcaTCP1) to 49.60 (DcaTCP2b) kDa, and the theoretical isoelectric point values ranged from 4.808 (DcaTCP2e) to 10.017 (DcaTCP7). The calculated grand average of hydrophobicity values ranged from −13.867 (DcaTCP2e) to 12.969 (DcaTCP1), indicating that most of the DcaTCP proteins were hydrophobic, except for DcaTCP2a, -2b, -2e, -5, -6, and -13 (Table 1).

### 2.2. Conserved Domain and Motif Analysis

**Figure 2:**
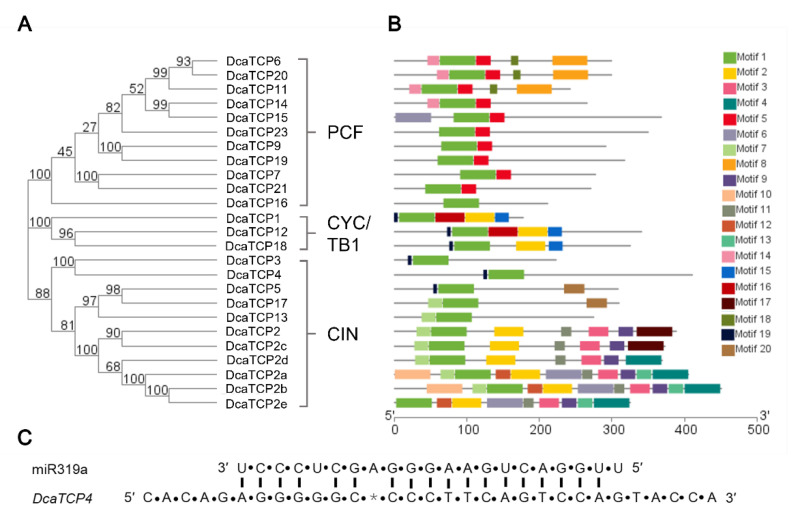
Genomic structure and motif composition of *D. catenatum* TCPs. (**A**) Phylogenetic tree of *D. catenatum* TCP proteins. (**B**) The conserved motifs in *D. catenatum* TCP proteins were identified using MEME. Each motif is represented with a specific color. (**C**) Alignment of putative target area for miR319a (aligned in reverse).

To better reveal the diversification among the *TCP* genes in *D. catenatum*, the DcaTCPs’ conserved motifs were analyzed. An NJ phylogenetic tree was constructed using the DcaTCP protein sequences (Figure 2A). The MEME online tool was used to predict the conversed motif compositions of the DcaTCPs (Figure 2B). The number of motifs varied from 1 to 11. Motif 1, as the TCP domain, was identified in all the DcaTCPs. In addition, other motifs were only present in members of specific subclades, such as Motifs 3, 9, and 11 in DcaTCP2, -2a, -2b, -2c, -2d, and -2e; Motifs 8, 14, and 18 in DcaTCP6, -11, and -20; Motif 15 in DcaTCP1, -12, and -18; and Motif 20 in DcaTCP5 and -17, suggesting that they have subclade-specific functions. In *Arabidopsis*, miR319a controls JA biosynthesis and leaf senescence by cleaving *TCP* transcription factors [14,20]. *AtTCP2*, *-3*, *-4*, *-10*, and *-24*, which belong to the CIN clade, are miR319a-targeted genes in *Arabidopsis*. miR319a is a conserved microRNA that regulates the expression of CIN subclade members [3,18,27]. Therefore, we speculated that the *DcaTCP* genes were miR319a targets in *D. catenatum*. miRNA-target complementarity analysis showed that *DcaTCP4,* a member of CIN family, might be a target gene of miR319a (Figure 2C).

### 2.3. Subcellular Localization

**Figure 3:**
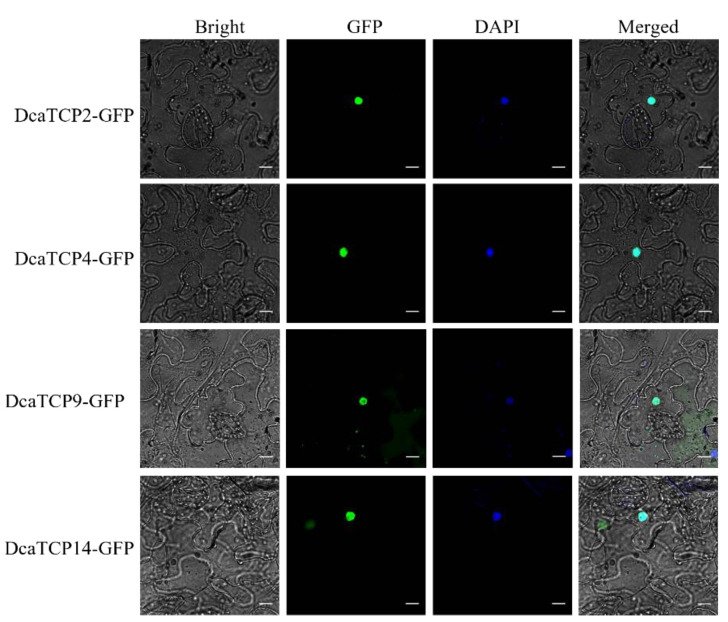
Subcellular localization of DcaTCP-GFP in *Nicotiana benthamiana* leaves. DcaTCP2-GFP, DcaTCP4-GFP, DcaTCP9-GFP and DcaTCP14-GFP were localized in the nucleus. GFP (Green Fluorescent Protein) is a versatile biological marker for visualizing protein localization. DAPI (4’,6-diamidino-2-phenylindole) is a blue-fluorescent DNA stain, which is used as a nuclear counterstain in fluorescence microscopy. Bar = 50 μm.

The known members of the *TCP* gene family function as transcription factors that regulate specific plant growth and developmental processes. Four *DcaTCPs* genes, *DcaTCP9* and *DcaTCP14* from Class I and *DcaTCP2* and *DcaTCP4* from Class II, were selected for the further analysis. The *GFP* gene was fused with each *DcaTCP* as a reporter. DcaTCP2, -4, -9, and -14 were all detected in nuclei (Figure 3), which suggests that they may function as transcription factors.

**Figure 4:**
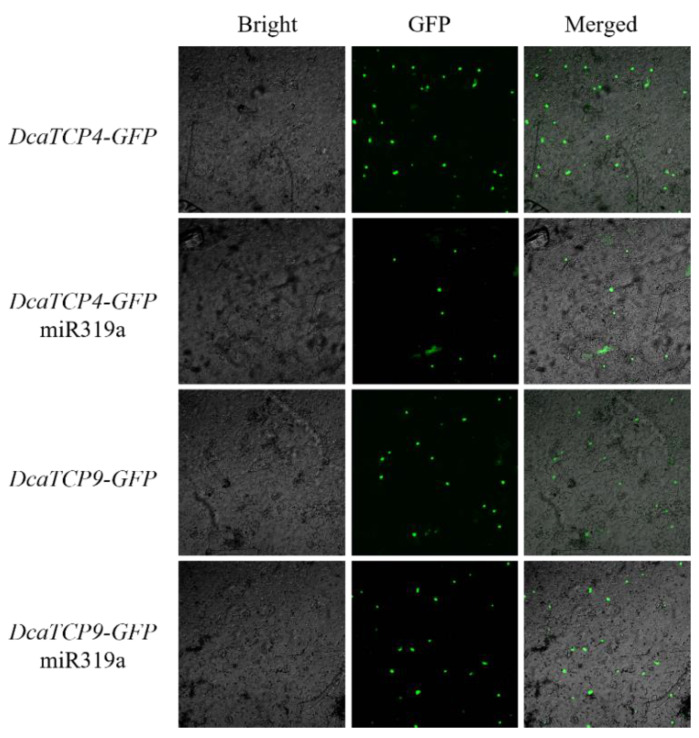
Complementarity analysis of miR319a and target genes. GFP (Green Fluorescent Protein) is a versatile biological marker. Bar = 50 μm.

miRNA-target complementarity analysis indicated that *DcaTCP4* may be a target gene of miR319a. To test this hypothesis, we co-overexpressed *DcaTCP4-GFP* with 35s:miR319a in accordance with the protocol of Liu [28]. The expression level of *DcaTCP4-GFP* co-injected with 35s:miR319a led to a significant downregulation compared with *DcaTCP4-GFP* alone (Figure 4). However, there was no obvious difference in expression between *DcaTCP9-GFP* and *DcaTCP9-GFP* with 35s:miR319a. Thus, miR319a appeared to recognize and cleave *DcaTCP4*, as in *Arabidopsis*.

### 2.4. Class I and II TCPs Antagonistically Regulate LOX2 Expression

**Figure 5:**
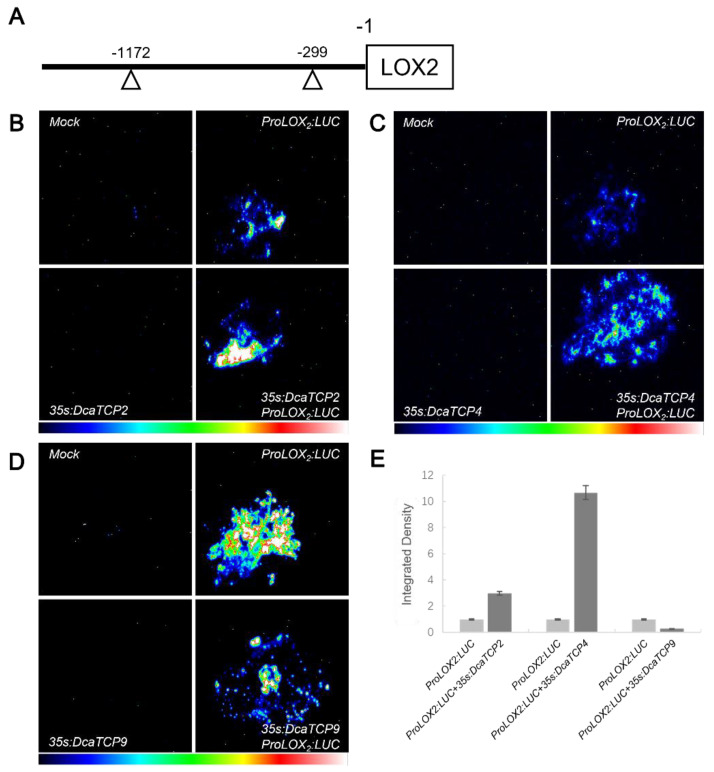
Transient expression analysis of DcaTCP2, DcaTCP4 and DcaTCP9 activities. (**A**) Schematic of the location of TCP-binding motif in the promoter of *LOX2* gene. (**B**) *LOX2* was activated by DcaTCP2. (**C**) *LOX2* was activated by DcaTCP4. (**D**) *LOX2* was repressed by DcaTCP9. (**E**) Quantification of relative luminescence intensities in (**B**–**D**) (mean ± SD, *n* = 5).

Class I and II TCP transcription factors in *Arabidopsis* have been reported to antagonistically control *LOX2* expression [29]. Consequently, we speculated that Class I and II TCP transcription factors in *D. catenatum* regulated *LOX2* expression by the same mechanism. To test this hypothesis, the *AtLOX2* promoter contained the TCP protein binding site (GTGGTCC and GGACCAC) (Figure 5A) was used to determine whether Class I (DcaTCP2 and DcaTCP4) and Class II (DcaTCP9) TCP transcription factors regulate the expression of the *AtLOX2* gene. When the *ProAtLOX2:LUC* reporter plasmid was co-transfected with the *35S:DcaTCP2* or *35S:DcaTCP4* effector plasmid, a strong LUC activity was detected. However, in the absence of the effector *35S:DcaTCP2* or *35S:DcaTCP4* plasmid, the LUC activity was much lower (Figure 5B,C,E). Thus, DcaTCP2 and DcaTCP4 indeed activated the transcription of *AtLOX2* in vivo. However, when the *ProAtLOX2:LUC* reporter plasmid was co-transfected with the *35S:DcaTCP9* effector plasmid, a weak LUC activity was detected, but in the absence of the effector *35S:DcaTCP9* plasmid, the LUC activity was much greater (Figure 5D,E). Thus, DcaTCP9 inhibited the transcription of *AtLOX2* in vivo. In conclusion, the Class I DcaTCPs (DcaTCP9) inhibited the expression of *AtLOX2*, and the Class II DcaTCPs (DcaTCP2 and DcaTCP4) activated *AtLOX2.*

### 2.5. Expression Profiles of TCP Genes in D. catenatum

Gene expression patterns are correlated with their functions [30]. To explore the possible functions of *DcaTCP* genes in *D. catenatum*, transcriptomic data were used to determine the expression changes of *DcaTCP* gene family members exposed to different phytohormone treatments.

**Figure 6:**
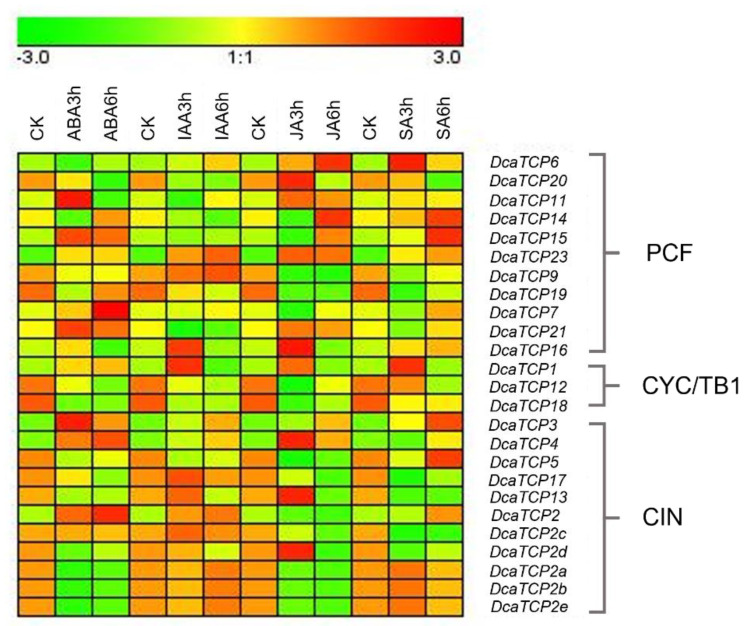
Expression analysis of *DcaTCP* genes after treatment with ABA, IAA, JA and SA. ABA: abscisic acid, IAA: indole-3-acetic acid, JA: jasmonic acid, SA: salicylic acid. ck: control check, 3 h: phytohormone treatments after 3 h, 6 h: phytohormone treatments after 6 h.

As shown in Figure 6, at 3 h after the JA treatment, the expression of *DcaTCP4* increased significantly, reaching the highest level, but the expression of *DcaTCP9* decreased. These results are consistent with antagonistic functions of Class I and II TCP proteins in the control of JA biosynthesis. *DcaTCP2* has six homologs in *D. catenatum*, and their expression levels varied. The expression of *DcaTCP2* increased in response to ABA and IAA treatments. The expression of *DcaTCP2d* increased after exposure to the JA treatment, but decreased after exposure to other phytohormone treatments. *DcaTCP2a*, -*2b*, and -*2e* had similar responses to the different phytohormone treatments. Thus, the *DcaTCP2* homologs appeared to have undergone functional differentiation during evolution.

## 3. Discussion

*D. catenatum* is the main component of several traditional medicines and health care products used to settle upset stomachs, promote body-fluid production, and nourish Yin [31,32]. The TCPs are crucial plant-specific transcription factors. The TCP transcription factors have been identified in many plant species. However, there are no reports on the TCP transcription factors in *D. catenatum.* Here, we identified 25 TCP members in the genome of the orchid *D. catenatum*. Using the sequences of 24 TCPs from *A. thaliana* and 25 TCPs from *D. catenatum*, we constructed a phylogenetic tree (Figure 1) and analyzed the phylogenetic relationships of *DcaTCP* genes (Figure 2A). To better understand the structural and functional features of DcaTCPs, 20 conserved motifs were investigated (Figure 2B), and the DcaTCP proteins within the same subclades shared similar motifs. These results might explain the functional redundancy that occurs among members of the same subclade.

Representative *DcaTCP* genes, *DcaTCP9* and *DcaTCP14* from Class I and *DcaTCP2* and *DcaTCP4* from Class II, were selected to analyze the functions of *DcaTCPs* in *D. catenatum.* They all localized in the nucleus (Figure 3), which demonstrated that the DcaTCPs might act as transcription factors to regulate plant growth and development. In *Arabidopsis*, Class I and II TCP proteins play opposite regulatory roles in the expression of *AtLOX2* by binding to its promoter. AtTCP4 activates the expression of *AtLOX2*, whereas AtTCP9 inhibits the expression of *AtLOX2* [20]. *AtLOX2* is a key enzyme in the JA-synthesis pathway, which regulates the synthesis of anthocyanins and the development of trichomes [33]. The antagonistic functions of Class I and II TCP proteins in the control of leaf development through the JA-signaling pathway have been documented previously [29]. Therefore, we investigated whether the functions of the two TCP subfamilies in *Dendrobium* were similar to those in *Arabidopsis*. DcaTCP2 and DcaTCP4 positively regulated the expression of *AtLOX2*, and DcaTC9 negatively regulated the expression of *AtLOX2* (Figure 5), which was consistent with the regulation in *Arabidopsis*. This indicated that *Dendrobium* and *Arabidopsis* shared the same TCP regulatory element. This result also suggested that the functions of the TCP transcription factors were conserved between monocotyledons and dicotyledons.

Phytohormones are important signaling substances in plants. They play important roles in plant defense, fertilization, and growth and development. The TCP transcription factors are involved in the phytohormone-signaling pathways in some plant species [34]. To determine the expression profiles and understand the roles of *DcaTCP* genes in response to phytohormones, transcriptomic data after exposure to ABA, IAA, JA and SA treatments were used. The expression levels of *DcaTCP* genes were affected by the different phytohormone treatments (Figure 6). This indicated that DcaTCP transcription factors in *D. catenatum* were involved in phytohormone responses, which supported their roles in regulating plant growth and development. The mechanism behind the *DcaTCP* genes’ participation in various phytohormone responses need further study.

## 4. Materials and Methods

### 4.1. Plant Material and Phytohormone Treatments

*Dendrobium catenatum* Lindl. was cultivated in a 5:1 (*w*/*w*) soil: sand mixture. In a greenhouse, the plants were maintained at 24 °C with 60–80% relative humidity and a 16 h photoperiod (daytime, 06:00–20:00).

35s:miR319a was kindly provided by Diqiu Yu [35]. miR319a was cloned into binary vector pOCA30 to generate construct 35s:miR319a. *ProLOX2:LUC* was kindly provided by Chuanyou Li [36]. The *LOX2* promoter was cloned into pENTR vector and then fused with the luciferase reporter binary vector pGWB35 using gateway reaction to generate the reporter construct *ProLOX2:LUC.*

Six-month-old plants were treated with 0.2 μM/L abscisic acid (ABA), 2 μM/L indole-3-acetic acid (IAA), 10 μM/L Jasmonate (JA) and 10 μM/L salicylic acid (SA) for 3 h or 6 h, respectively. Non-treatment plants were used as the control. Then, the samples were quickly frozen in liquid nitrogen and stored at −80 °C until use.

### 4.2. Identification of TCP Family Genes in D. catenatum

Data for *D. catenatum* proteins were downloaded from the *D. catenatum* Genome (http://orchidbase.itps.ncku.edu.tw/est/Dendrobium_2019.aspx, accessed on 5 June 2021). Using the following pipeline, TCP-like sequences were identified from publicly available *D. catenatum* sequences. First, the complete coding DNA sequences (CDSs) of 24 *TCP* genes from *A. thaliana* were extracted from the public database TAIR (https://www.arabidopsis.org/, accessed on 12 June 2021) (accession listed in Table S1), using local BLAST algorithm-based searches and a hidden Markov model (HMM) to identify the *TCP* genes [5,37]. Local BLASTN and BLASTP searches were conducted with known AtTCP sequences as the query to search the genome sequences of *D. catenatum*. These searches identified the initial candidate genes containing putative TCP domains in *D. catenatum*. The HMM profile (accession number PF03634) from the Pfam protein family database (http://pfam.sanger.ac.uk, accessed on 9 July 2021) was applied to confirm the presence of the conserved TCP domain in each candidate TCP protein [38]. Finally, the identified TCP-like sequences were double-checked for the presence of a functional TCP domain/motif using the NCBI Conserved Domains tool [39]. The retrieved sequences lacking a TCP domain were removed prior to further analyses.

### 4.3. Conserved Motif Analysis

The Multiple Em for Motif Elucidation (MEME) program (http://meme-suite.org/index.html, accessed on 16 July 2021) was used to analyze the conserved motifs of DcaTCP proteins, with the following site distribution parameters: (1) the optimum motif width was set from 6 to 50; and (2) the maximum number of motifs to identify was set as 20. Additionally, each sequence that appeared at least once was used [40].

### 4.4. Sequence Characterization and Phylogenetic Analysis

LaserGene7 was used to further analyze the identified DcaTCPs, including their CDS lengths, protein sizes, protein molecular weights, isoelectric points, and grand averages of hydropathicity. All the amino acid sequence-based phylogenetic trees were constructed using the MEGA7.0 program, whereas the protein-based phylogenetic tree was constructed using the Neighbor-Joining (NJ) method with a bootstrap value of 1000 to test the reliability [41].

### 4.5. Subcellular Localization and miRNA-Target Interactions

The CDSs of *DcaTCP2*, *-4*, *-9*, and *-14* were amplified using a Phanta^®^ Max Super-Fidelity DNA Polymerase (Vazyme Biotech Co., Ltd., Nanjing, China) with relative primers (listed in Table S2), then using ClonExpress^®^ II (Vazyme Biotech Co., Ltd., Nanjing, China) inserted into the *pRI101-GFP* vector to allow its sequencing-based validation. The *35S:GFP-DcaTCP2, -4, -9, and -14* were transferred into *Agrobacterium tumefaciens* EHA105 using electroporation and then injected into *N. benthamiana* leaves, respectively. The penetrated plants were cultivated for 3 days. Laser confocal microscopy (Olympus Optical Co., Ltd., Tokyo, Japan) was performed in accordance with Du [42].

miRNA319a-target interactions were analyzed according to Liu [28]. 35s:miR319a was introduced into *Agrobacterium* GV3101-pSoup and then co-injected with *DcaTCP4-GFP* and *DcaTCP9-GFP* into *N. benthamiana* leaves, separately.

### 4.6. Transient Expression in Nicotiana benthamiana

Transactivation activity detection assays were performed using *35S:GFP-DcaTCP2, -4, -9* as effector and *ProLOX2:LUC* as reporter [43]. *35S:GFP-DcaTCP2, -4* and *-9* co-injected with *ProLOX2:LUC* into *N. benthamiana* leaves, respectively. The penetrated plants were cultivated for 3 days. Then, 1 mM luciferin was sprayed onto the injected *N. benthamiana* leaves, which were then placed in the dark for 3 min. Luciferase luminescence was captured using a Tanon-5200 Chemiluminescent Imaging System (Tanon Science & Technology Co., Ltd., Shanghai, China) automated chemiluminescence image analysis system with a low-light cooled CCD camera.

### 4.7. Transcriptomic Data

Transcriptomic data from phytohormone-treated *D. catenatum* were obtained from the Biodiversity Data Center (https://data.iflora.cn/Home/DataContent?data_gd=89131009-82d9-ad4b-faaa-1bad087095e2, accessed on 19 August 2021). The *DcaTCP* genes’ expression levels were analyzed with moderated t-statistics using deseq2, and fold-change values were also calculated [44]. A heatmap was constructed using Genesis.

## 5. Conclusions

Our study provided the first genome-wide analysis of the *TCP* gene family in *D. catenatum*. It revealed the difference of *DcaTCP* gene function in Class I and Class II. The expression of 25 *DcaTCP* in response to various phytohormones treatments was characterized. The results of this study lay the basis for further research on the functions of the *TCP* gene family members in growth and development process, which will promote their application in *D. catenatum.*

## Figures and Tables

**Table 1:** Identification and characteristics of *TCP* genes in *D. catenatum*.

Gene Name	Accession Number	CDS Length (bp)	Protein Size (aa)	MW (kD)	PI	GRAVY
DenTCP1	XP_028548590	537	178	20.03	9.809	12.969
DenTCP2	XP_020673786	1170	389	42.07	8.417	5.644
DenTCP2a	XP_028551220	1221	406	44.45	6.723	−1.370
DenTCP2b	XP_020679251	1353	450	49.60	6.875	−0.761
DenTCP2c	XP_020703327	1122	373	40.56	8.798	7.145
DenTCP2d	XP_020675099	1110	369	40.64	8.402	3.993
DenTCP2e	XP_028551222	978	325	35.19	4.808	−13.867
DenTCP3	XP_028553615	672	223	24.98	9.531	6.732
DenTCP4	XP_020674146	1236	411	45.23	9.346	9.242
DenTCP5	XP_028553819	927	308	34.67	6.765	−0.807
DenTCP6	XP_028550088	903	300	31.82	6.740	−1.591
DenTCP7	XP_020688226	837	278	29.78	10.017	9.840
DenTCP9	XP_020701159	879	292	30.26	9.460	5.574
DenTCP11	XP_020699509	732	243	25.64	7.132	0.397
DenTCP12	XP_028556330	1026	341	37.73	7.472	1.493
DenTCP13	XP_020702002	828	275	30.77	6.019	−3.960
DenTCP14	XP_020695302	801	266	28.62	8.720	3.578
DenTCP15	XP_020697754	1107	368	38.87	8.131	4.875
DenTCP16	XP_020686130	636	211	22.32	8.154	1.389
DenTCP17	XP_020702494	933	310	35.05	7.097	0.265
DenTCP18	XP_028550169	978	325	36.22	7.172	0.662
DenTCP19	XP_020673638	954	317	33.20	8.933	3.916
DenTCP20	XP_020693581	900	299	31.79	8.204	2.740
DenTCP21	XP_020682551	816	271	27.21	9.723	6.533
DenTCP23	XP_020696150	1053	350	35.96	9.514	6.902

## Data Availability

Not applicable.

## Supplementary Materials

The following are available online at https://www.mdpi.com/article/10.3390/ijms221910269/s1.

## References

[1] Doebley J., Stec A., Hubbard L. (1997). The evolution of apical dominance in maize. Nature.

[2] Luo D., Carpenter R., Vincent C., Copsey L., Coen E. (1996). Origin of floral asymmetry in Antirrhinum. Nature.

[3] Cubas P., Lauter N., Doebley J., Coen E. (1999). The TCP domain: A motif found in proteins regulating plant growth and development. Plant J..

[4] Kosugi S., Ohashi Y. (1997). PCF1 and PCF2 specifically bind to cis elements in the rice proliferating cell nuclear antigen gene. Plant Cell.

[5] Martin-Trillo M., Cubas P. (2010). TCP genes: A family snapshot ten years later. Trends Plant Sci..

[6] Kosugi S., Ohashi Y. (2002). DNA binding and dimerization specificity and potential targets for the TCP protein family. Plant J..

[7] Navaud O., Dabos P., Carnus E., Tremousaygue D., Herve C. (2007). TCP transcription factors predate the emergence of land plants. J. Mol. Evol..

[8] Parapunova V., Busscher M., Busscher-Lange J., Lammers M., Karlova R., Bovy A.G., Angenent G.C., de Maagd R.A. (2014). Identification, cloning and characterization of the tomato TCP transcription factor family. BMC Plant Biol..

[9] Ma X., Ma J., Fan D., Li C., Jiang Y., Luo K. (2016). Genome-wide Identification of TCP Family Transcription Factors from Populus euphratica and Their Involvement in Leaf Shape Regulation. Sci. Rep..

[10] Shi P., Guy K.M., Wu W., Fang B., Yang J., Zhang M., Hu Z. (2016). Genome-wide identification and expression analysis of the *ClTCP* transcription factors in *Citrullus lanatus*. BMC Plant Biol..

[11] Zhou Y., Xu Z., Zhao K., Yang W., Cheng T., Wang J., Zhang Q. (2016). Genome-Wide Identification, Characterization and Expression Analysis of the *TCP* Gene Family in *Prunus mume*. Front. Plant Sci..

[12] Zhang S.T., Zhou Q., Chen F., Wu L., Liu B.J., Li F., Zhang J.Q., Bao M.Z., Liu G.F. (2020). Genome-Wide Identification, Characterization and Expression Analysis of TCP Transcription Factors in Petunia. Int. J. Mol. Sci..

[13] Ling L., Zhang W., An Y., Du B., Wang D., Guo C. (2020). Genome-wide analysis of the *TCP* transcription factor genes in five legume genomes and their response to salt and drought stresses. Funct. Integr. Genom..

[14] Nag A., King S., Jack T. (2009). miR319a targeting of *TCP4* is critical for petal growth and development in *Arabidopsis*. Proc. Natl. Acad. Sci. USA.

[15] Aguilar-Martínez J.A., Poza-Carrión C., Cubas P. (2007). *Arabidopsis BRANCHED1* Acts as an Integrator of Branching Signals within Axillary Buds. Plant Cell.

[16] Poza-Carrión C., Aguilar-Martínez J.A., Cubas P. (2007). Role of *TCP* Gene *BRANCHED1* in the Control of Shoot Branching in *Arabidopsis*. Plant Signal. Behav..

[17] Efroni I., Blum E., Goldshmidt A., Eshed Y. (2008). A protracted and dynamic maturation schedule underlies *Arabidopsis* leaf development. Plant Cell.

[18] Palatnik J.F., Allen E., Wu X., Schommer C., Schwab R., Carrington J.C., Weigel D. (2003). Control of leaf morphogenesis by microRNAs. Nat. Genet..

[19] Koyama T., Mitsuda N., Seki M., Shinozaki K., Ohme-Takagi M. (2010). TCP Transcription Factors Regulate the Activities of ASYMMETRIC LEAVES1 and miR164, as Well as the Auxin Response, during Differentiation of Leaves in *Arabidopsis*. Plant Cell.

[20] Schommer C., Palatnik J.F., Aggarwal P., Chetelat A., Cubas P., Farmer E.E., Nath U., Weigel D. (2008). Control of jasmonate biosynthesis and senescence by miR319 targets. PLoS Biol..

[21] Zhao F., Wang C., Han J., Zhu X., Li X., Wang X., Fang J. (2017). Characterization of miRNAs responsive to exogenous ethylene in grapevine berries at whole genome level. Funct. Integr. Genom..

[22] Welchen E., Gonzalez D.H. (2006). Overrepresentation of elements recognized by TCP-domain transcription factors in the upstream regions of nuclear genes encoding components of the mitochondrial oxidative phosphorylation Machinery. Plant Physiol..

[23] Qi X., Qu Y., Gao R., Jiang J., Fang W., Guan Z., Zhang F., Zhao S., Chen S., Chen F. (2019). The Heterologous Expression of a Chrysanthemum nankingense TCP Transcription Factor Blocks Cell Division in Yeast and *Arabidopsis thaliana*. Int. J. Mol. Sci..

[24] Lan J.Q., Qin G.J. (2020). The Regulation of CIN-like TCP Transcription Factors. Int. J. Mol. Sci..

[25] Hou B., Luo J., Zhang Y., Niu Z., Xue Q., Ding X. (2017). Iteration expansion and regional evolution: Phylogeography of *Dendrobium officinale* and four related taxa in southern China. Sci. Rep..

[26] Lei Z., Zhou C., Ji X., Wei G., Huang Y., Yu W., Luo Y., Qiu Y. (2018). Transcriptome Analysis Reveals genes involved in flavonoid biosynthesis and accumulation in *Dendrobium catenatum* From Different Locations. Sci. Rep..

[27] Palatnik J.F., Wollmann H., Schommer C., Schwab R., Boisbouvier J., Rodriguez R., Warthmann N., Allen E., Dezulian T., Huson D. (2007). Sequence and expression differences underlie functional specialization of *Arabidopsis* microRNAs miR159 and miR319. Dev. Cell.

[28] Liu Q., Wang F., Axtell M.J. (2014). Analysis of Complementarity Requirements for Plant MicroRNA Targeting Using a *Nicotiana benthamiana* Quantitative Transient Assay. Plant Cell.

[29] Danisman S., van der Wal F., Dhondt S., Waites R., de Folter S., Bimbo A., van Dijk A.-J., Muino J.M., Cutri L., Dornelas M.C. (2012). *Arabidopsis* Class I and Class II TCP Transcription Factors Regulate Jasmonic Acid Metabolism and Leaf Development Antagonistically. Plant Physiol..

[30] Xu Z.D., Sun L.D., Zhou Y.Z., Yang W.R., Cheng T.R., Wang J., Zhang Q.X. (2015). Identification and expression analysis of the SQUAMOSA promoter-binding protein (SBP)-box gene family in *Prunus mume*. Mol. Genet. Genom..

[31] Yan L., Wang X., Liu H., Tian Y., Lian J., Yang R., Hao S., Wang X., Yang S., Li Q. (2015). The Genome of *Dendrobium officinale* Illuminates the Biology of the Important Traditional Chinese Orchid Herb. Mol. Plant.

[32] Tang H., Zhao T., Sheng Y., Zheng T., Fu L., Zhang Y. (2017). *Dendrobium officinale* Kimura et Migo: A Review on Its Ethnopharmacology, Phytochemistry, Pharmacology, and Industrialization. Evid. Based Complement. Altern. Med..

[33] Vick B.A., Zimmerman D.C. (1983). The biosynthesis of jasmonic acid—A physiological-role for plant lipoxygenase. Biochem. Biophys. Res. Commun..

[34] Feng Z.J., Xu S.C., Liu N., Zhang G.W., Hu Q.Z., Gong Y.M. (2018). Soybean TCP transcription factors: Evolution, classification, protein interaction and stress and hormone responsiveness. Plant Physiol..

[35] Liang G., He H., Li Y., Yu D.Q. (2012). A new strategy for construction of artificial miRNA vectors in *Arabidopsis*. Planta.

[36] Zhai Q.Z., Yan L.H., Tan D., Chen R., Sun J.Q., Gao L.Y., Dong M.Q., Wang Y.C., Li C.Y. (2013). Phosphorylation-Coupled Proteolysis of the Transcription Factor MYC2 Is Important for Jasmonate-Signaled Plant Immunity. PLoS Genet..

[37] Jin J.P., Tian F., Yang D.C., Meng Y.Q., Kong L., Luo J.C., Gao G. (2017). PlantTFDB 4.0: Toward a central hub for transcription factors and regulatory interactions in plants. Nucleic Acids Res..

[38] Finn R.D., Alex B., Jody C., Penelope C., Eberhardt R.Y., Eddy S.R., Andreas H., Kirstie H., Liisa H., Jaina M. (2014). Pfam: The protein families database. Nucleic Acids Res..

[39] Lu S., Wang J., Chitsaz F., Derbyshire M.K., Geer R.C., Gonzales N.R., Gwadz M., Hurwitz D.I., Marchler G.H., Song J.S. (2020). CDD/SPARCLE: The conserved domain database in 2020. Nucleic Acids Res..

[40] Bailey T.L., Johnson J., Grant C.E., Noble W.S. (2015). The MEME Suite. Nucleic Acids Res..

[41] Kumar S., Stecher G., Tamura K. (2016). MEGA7: Molecular Evolutionary Genetics Analysis Version 7.0 for Bigger Datasets. Mol. Biol. Evol..

[42] Du J., Hu S., Yu Q., Wang C., Yang Y., Sun H., Yang Y., Sun X. (2017). Genome-Wide Identification and Characterization of *BrrTCP* Transcription Factors in *Brassica rapa* ssp. rapa. Front. Plant Sci..

[43] Chen H., Zou Y., Shang Y., Lin H., Wang Y., Cai R., Tang X., Zhou J.-M. (2008). Firefly luciferase complementation imaging assay for protein-protein interactions in plants. Plant Physiol..

[44] Anders S., Huber W. (2010). Differential expression analysis for sequence count data. Genome Biol..

