# Supplementary material for: Genome-Wide Analysis of the TCP Transcription Factor Genes in Dendrobium catenatum Lindl
Source: Int J Mol Sci. 2021 Sep 24;22(19):10269. doi: 10.3390/ijms221910269 (PMC8508941; doi:10.3390/ijms221910269)
Supplement: Supplementary file 1 [file ijms-22-10269-s001.zip › ijms-1374824-supplementary.pdf]

Table S1. The accession of *AtTCP* gene.

**Gene**	**Accession**
AtTCP1	AT1G67260
AtTCP2	AT4G18390
AtTCP3	AT1G53230
AtTCP4	AT3G15030
AtTCP5	AT5G60970
AtTCP6	AT5G41030
AtTCP7	AT5G23280
AtTCP8	AT1G58100
AtTCP9	AT2G45680
AtTCP10	AT2G31070
AtTCP11	AT2G37000
AtTCP12	AT1G68800
AtTCP13	AT3G02150
AtTCP14	AT3G47620
AtTCP15	AT1G69690
AtTCP16	AT3G45150
AtTCP17	AT5G08070
AtTCP18	AT3G18550
AtTCP19	AT5G51910
AtTCP20	AT3G27010
AtTCP21	AT5G08330
AtTCP22	AT1G72010
AtTCP23	AT1G35560
AtTCP24	AT1G30210

Table S2. The primer sequences of *DcaTCPs'* CDS.

**Primer Name**	**Sequence (5' to 3')**
DcaTCP2-SalI-F	*GCAGCGGCCGTCGAC*ATGGAGGTGAAACAACAAGC
DcaTCP2-EcoRI-R	*GTTGATTCAGAATTC*TCAGCTTTTTCTCTTTCC
DcaTCP4-SalI-F	*GCAGCGGCCGTCGAC*ATGGCCAAATATTTTCGGAG
DcaTCP4-EcoRI-R	*GTTGATTCAGAATTC*CTAGTAATGAGAAGCAGAGG
DcaTCP9-SalI-F	*GCAGCGGCCGTCGAC*ATGGATTTCCAGGCCAAGAG
DcaTCP9-EcoRI-R	*GTTGATTCAGAATTC*CTAAGCCGCTTCTTTTACCG
DcaTCP14-SalI-F	*GCAGCGGCCGTCGAC*ATGGAGGGAGAAAACATTG
DcaTCP14-EcoRI-R	*GTTGATTCAGAATTC*CTAGGAGTCGCTTGTGCTC
